# Complete Genome Sequence of Fusobacterium necrophorum subsp. *necrophorum* ATCC 25286

**DOI:** 10.1128/MRA.00025-19

**Published:** 2019-02-21

**Authors:** Ariana Umaña, Justin A. Lemkul, Daniel J. Slade

**Affiliations:** a Department of Biochemistry, Virginia Polytechnic Institute and State University, Blacksburg, Virginia, USA; University of Arizona

## Abstract

Fusobacterium necrophorum is a pathogenic Gram-negative, anaerobic bacterium. In this study, we present the first complete genome sequence of Fusobacterium necrophorum subsp. necrophorum ATCC 25286. These data provide a critical advancement in our understanding of virulence factors that could contribute to F. necrophorum pathogenesis in both human and livestock infections.

## Abstract (precis)

Fusobacterium necrophorum is a pathogenic Gram-negative, anaerobic bacterium. In this study, we present the first complete genome sequence of Fusobacterium necrophorum subsp.

## ANNOUNCEMENT

*Fusobacterium* is a genus of anaerobic bacteria that cause serious and even fatal infections of cattle, sheep, and humans. Recent research has uncovered that Fusobacterium nucleatum is overrepresented in colonic tumors, and this bacterium can drive the progression and severity of colorectal cancer (1, 2). In humans, Fusobacterium necrophorum can cause the rare and life-threatening Lemierre syndrome, which begins as bacterial pharyngitis and rapidly progresses to septic thrombophlebitis of the jugular vein (3). Lemierre syndrome most frequently manifests in patients age 15 to 30 and can lead to further complications, including pharyngeal and lung abscesses (4). F. necrophorum have been classified into F. necrophorum subsp. necrophorum (biotype A) and F. necrophorum subsp. funduliforme (biotype B) (5). Fusobacterium necrophorum subsp. funduliforme is most commonly the cause of human infection, with Fusobacterium necrophorum subsp. necrophorum being the predominant animal pathogen. In cattle and sheep, biotype A is primarily responsible for liver abscesses and footrot (necrotic pododermatitis), for which antibiotics are an effective treatment (6).

In this study, we present the first complete genome sequence of Fusobacterium necrophorum subsp. necrophorum ATCC 25286 (5). The genome consists of one 2,678,415-bp circular chromosome (G+C content of 34%) and no plasmids. Annotation detected 2,343 protein open reading frames, 18 rRNAs, and 51 tRNAs. In addition, a 66-kb prophage region (75 protein-encoding genes; genomic position 477708 to 543489) was discovered, with the greatest similarity to a phage from the genus *Clostridium* identified. We have identified the *lktBAC* leukotoxin gene cluster and also report an increase in the number of type 5a-secreted autotransporter virulence factors compared with an annotation from the previous draft genome (87 contigs; NCBI BioProject accession number PRJNA257880).

F. necrophorum subsp. necrophorum ATCC 25286 was purchased through the ATCC. Bacteria from a single colony were grown in Columbia Broth, supplemented with hemin (5 µg/mL) and menadione (0.5 µg/mL) (CBHK) to stationary phase at 37°C in an anaerobic chamber (90% N_2_, 5% CO_2_, and 5% H_2_). Genomic DNA was isolated in deionized water (diH_2_O) using a Wizard isolation kit (Promega) and was quantitated using a Qubit fluorimeter (Life Technologies, Inc.). Long-read sequences were acquired by preparing 500 ng of DNA with a rapid sequencing kit (catalog number SQK-RAD004; Oxford Nanopore Technologies) and sequencing using a SpotON flow cell (R9.4) and a MinION sequencer (Oxford Nanopore Technologies). MinKnow 3.1.8 software was used to acquire raw data and basecall DNA sequences. Sequencing adapters from Oxford Nanopore reads were removed with Porechop (7), and a total of 71,386 reads (mean read length, 1,731 bp; maximum read length, 91,983 bp) were used for genome assembly. The same DNA preparation used for MinION sequencing was used for short-read DNA sequencing on a MiSeq nano instrument at the Genomic Sequence Center at the Virginia Tech Biocomplexity Institute. Briefly, 150 ng of genomic DNA was fragmented to 400 bp using a Covaris M220 focused ultrasonicator. The ends were repaired, and an A base was added to the 3′ end for ligation to the adapters, which have a single T base overhang at their 3′ end. Following ligation, the libraries were amplified by 7 cycles of PCR and barcoded. The library generated was validated by the use of an Agilent TapeStation and quantitated using a Quant-iT double-stranded (dsDNA) high sensitivity (HS) kit (Invitrogen) and quantitative PCR (qPCR). The libraries were then pooled and sequenced using a MiSeq nano instrument. In summary, 999,276 reads at 150 bp each (150 mb total; 56× genome coverage) were used for genome assembly. The F. necrophorum ATCC 25286 genome was assembled using the open-source software package Unicycler under default parameters, version 0.4.3 (8), using our previously described pipeline (9). For Illumina and MinION data, the mean depth of coverage for the genome was 56× and 53×, respectively. Prokka 1.13 under default parameters was used to annotate genes (10). Potential integrated phage were detected using PHASTER (11). Annotation in GenBank was carried out by the NCBI Prokaryotic Genome Annotation Pipeline (12).

### Data availability.

The complete sequence and all raw data for the Fusobacterium necrophorum subsp. necrophorum ATCC 25286 genome have been deposited in NCBI under the accession numbers CP034842 (GenBank), PRJNA513186 (BioProject), SAMN10697414 (BioSample), and SRR8400822 and SRR8400823 (SRA raw sequencing reads). In addition, all data for this genome and additional bioinformatic analysis will be freely available on the FusoPortal (13) data repository of *Fusobacterium* genomes (http://fusoportal.org) and our Open Science Framework site (https://osf.io/2c8pv/). The deposited data are the first version of this genome.
